# Comparison of bivalirudin versus heparin in percutaneous coronary intervention for patients with acute coronary syndrome: A meta-analysis

**DOI:** 10.1097/MD.0000000000050046

**Published:** 2026-09-25

**Authors:** Chen Yang, Zhen Ning, Weiming Liang, Yuanming Liao, Yanzi Mai, Jie Li

**Affiliations:** a Department of Cardiovascular Medicine, The First Affiliated Hospital of Guangxi University of Science and Technology, Guangxi University of Science and Technology, Liuzhou, Guangxi Province, China.

**Keywords:** acute coronary syndrome, bivalirudin, heparin, meta-analysis, percutaneous coronary intervention

## Abstract

**Background:**

Bivalirudin and heparin are commonly employed anticoagulants in percutaneous coronary intervention (PCI) for patients diagnosed with acute coronary syndrome (ACS). This meta-analysis aimed to compare the safety and efficacy of bivalirudin and heparin in patients with ACS undergoing PCI.

**Methods:**

We systematically searched PubMed, Embase, Web of Science, and the Cochrane Library for randomized controlled trials (RCTs) and cohort studies comparing bivalirudin with heparin in patients with ACS undergoing PCI. The outcomes included all-cause mortality, cardiovascular mortality, major bleeding, major adverse cardiovascular events (MACEs), net adverse clinical events, myocardial infarction (MI), stent thrombosis (ST), and stroke.

**Results:**

This study included 11 RCTs and 17 cohort studies, encompassing 584,249 patients. Among in-hospital outcomes, bivalirudin was found to significantly reduce the incidence of all-cause mortality (relative risk [RR] = 0.86, 95% confidence interval [CI]: 0.84–0.88, *P* < .001, *I*^2^ = 0%) and major bleeding (RR = 0.74, 95% CI: 0.62–0.88, *P* < .001, *I*^2^ = 51%) compared with heparin, while there were no significant differences in MACEs, MI, ST, and stroke. Among 30-day follow-up outcomes, bivalirudin was found to significantly reduce the incidence of all-cause mortality (RR = 0.85, 95% CI: 0.76–0.95, *P* = .006, *I*^2^ = 24%), cardiovascular mortality (RR = 0.81, 95% CI: 0.70–0.94, *P* = .004, *I*^2^ = 1%), major bleeding (RR = 0.59, 95% CI: 0.49–0.72, *P* < .001, *I*^2^ = 67%), and net adverse clinical events (RR = 0.79, 95% CI: 0.70–0.89, *P* < .001, *I*^2^ = 55%) compared with heparin, while there were no significant differences in MACEs, MI, ST, and stroke.

**Conclusion:**

This meta-analysis suggests that bivalirudin may offer advantages over heparin in reducing in-hospital and 30-day mortality and major bleeding in patients with ACS undergoing PCI, with no significant differences in ischemic outcomes such as MACEs, MI, ST, or stroke. These findings suggest that bivalirudin may be a reasonable alternative to heparin in selected patients, but more large-scale, long-term RCTs are needed to confirm these results.

## 1. Introduction

Coronary artery disease remains a critical global public health burden, not only afflicting populations in the UK and the USA but also posing widespread challenges across the world.^[1]^ Acute coronary syndrome (ACS), a spectrum encompassing ST-elevation myocardial infarction (STEMI), non-ST-elevation myocardial infarction (NSTEMI), and unstable angina (UA), is a leading cause of morbidity and mortality worldwide.^[2]^ Percutaneous coronary intervention (PCI) is a cornerstone treatment, with anticoagulation playing a critical role in balancing ischemic and bleeding risks. Heparin and bivalirudin are commonly used anticoagulants during PCI. However, previous studies have yielded conflicting results regarding their comparative safety and efficacy.

A prior meta-analysis by Al-Abdouh et al^[3]^ included only 10 trials, limiting statistical power for infrequent outcomes and precluding robust evaluation of safety endpoints. The meta-analysis by Zhai et al^[4]^ included only randomized controlled trials (RCTs; 70,199 patients), which may limit the generalizability of its findings to routine clinical practice. Moreover, this meta-analysis did not report net adverse clinical events (NACE) – a key composite endpoint balancing ischemic and bleeding risks.^[5]^ The meta-analysis by Liu et al^[6]^ was restricted exclusively to patients with STEMI and did not cover the broader ACS spectrum (including NSTEMI and UA), thereby limiting its generalizability to the entire ACS population undergoing PCI. Furthermore, given its publication year of 2020, the data included in that meta-analysis are relatively outdated.

To address the limitations of prior meta-analyses, we aimed to conduct an updated meta-analysis with a broader search strategy, a larger sample size, and separate analyses by study design (RCT or cohort) to provide more reliable evidence on the comparative safety and efficacy of bivalirudin and heparin in patients with ACS undergoing PCI. Therefore, the primary research hypothesis of this study was that bivalirudin, compared with heparin, reduces major bleeding and mortality without increasing ischemic events in ACS patients undergoing PCI.

## 2. Materials and methods

### 2.1. Protocol and registration

This meta-analysis was performed in adherence to the Preferred Reporting Items for Systematic Reviews and Meta-Analyses 2020 statement.^[7]^ The study protocol was prospectively registered in the International Prospective Register of Systematic Reviews (PROSPERO) prior to the initiation of the literature screening, with the unique registration number CRD420251139290. As this study is a systematic review and meta-analysis of previously published aggregate data, it did not involve direct patient enrollment, intervention, or collection of primary data. Therefore, ethical approval from an institutional review board and informed consent were not required.

### 2.2. Study search and selection criteria

This study carried out systematic and comprehensive literature retrieval in 4 electronic databases, including PubMed, Embase, Web of Science, and the Cochrane Library. The literature search was conducted up to August 8, 2025. The search approach adhered to the PICOS framework and utilized a combination of medical subject headings keywords and unconstrained textual phrases. The core retrieval keywords were defined as follows: “bivalirudin,” “heparin,” “acute coronary syndrome,” and “percutaneous coronary intervention.” The Boolean logic for the search strategy was set as follows: (bivalirudin) AND (heparin) AND (acute coronary syndrome) AND (percutaneous coronary intervention). Tables S1, S2, S3 and S4, Supplemental Digital Content 1, display details of the retrieved records across the 4 databases. After the initial retrieval, 2 independent reviewers further screened the retrieved records (including titles, abstracts, and full texts, where necessary) to ensure the accuracy of the included studies.

The inclusion criteria were as follows: patients with a clinical diagnosis of ACS, including STEMI, NSTEMI, and UA, who were planned to undergo PCI; patients in the intervention group treated with bivalirudin; patients in the control group treated with heparin; studies reporting at least one of the following prespecified outcome measures: all-cause mortality, cardiovascular mortality, major bleeding, major adverse cardiovascular events (MACEs), NACE, myocardial infarction (MI), stent thrombosis (ST), and stroke; and study design: RCT, prospective, or retrospective study.

The exclusion criteria were as follows: non-original research articles, including case reports, study protocols, letters, editorials, commentaries, reviews, and meta-analyses; irrelevance to the research topic (population not consisting of ACS patients); studies not comparing bivalirudin with heparin; overlapping patient cohorts; unavailable or non-extractable data; and absence of relevant outcome measures.

### 2.3. Data extraction and risk-of-bias assessment

Two independent researchers screened the retrieved records against predefined inclusion/exclusion criteria, extracted data (first author, publication year, patient baseline characteristics, group sample sizes, follow-up duration, main outcome definitions, and results), and cross-verified the data. Discrepancies were resolved via discussion or consultation with a third senior investigator.

The main outcomes include all-cause mortality, cardiovascular mortality, major bleeding, MACEs, NACE, MI, ST, or stroke. All-cause mortality – defined as death from any cause. Cardiovascular mortality – defined as death directly attributable to a cardiovascular etiology, with suggested subcategories including acute MI, sudden cardiac death, heart failure, stroke, cardiovascular procedure, cardiovascular hemorrhage, and other cardiovascular causes. Major bleeding – defined as TIMI major bleeding or BARC (Bleeding Academic Research Consortium) type 3 to 5 bleeding, consistent with standardized bleeding endpoint definitions for cardiovascular clinical trials. MACEs – defined as the composite of cardiovascular death, MI, and stroke. NACE – defined as the composite of major bleeding or MACEs. MI – defined using the Third Universal Definition of Myocardial Infarction criteria, with diagnostic thresholds based on the 99th percentile upper reference limit for cardiac biomarkers and supporting evidence from new ischemic ECG changes or imaging findings of new loss of viable myocardium or new regional wall motion abnormality. ST – defined according to the Academic Research Consortium (ARC) criteria as definite, probable, or possible ST, with timing classification into acute (<24 hours), subacute (24 hours to 30 days), late (1 month to 1 year), and very late (>1 year). Definite ST requires angiographic or pathological demonstration of thrombus in the stent region accompanied by acute ischemic symptoms, new ischemic ECG changes, or typical rise and fall in cardiac biomarkers. Stroke – defined as the rapid onset of a new, persistent neurological deficit lasting at least 24 hours (or leading to death before 24 hours).^[2,5,8,9]^ The standardized outcome definitions presented above were developed a priori based on established cardiovascular endpoint consensus documents (ARC-2, ACC/AHA key data elements, and BARC criteria). These definitions were used as reference standards during data extraction. For each included study, we extracted outcome events that most closely matched these prespecified definitions.

The quality of the included studies was independently evaluated by 2 reviewers. For retrospective and prospective studies, assessment was performed using the Newcastle–Ottawa Scale (NOS),^[10]^ which comprises 8 items: representativeness of the exposed cohort; selection of the nonexposed cohort; ascertainment of exposure; demonstration that the outcome of interest was absent at baseline; comparability of cohorts; assessment of outcome; the duration of the follow-up visit was sufficient to observe the occurrence of the outcome event; and adequacy of follow-up in cohort studies. The Cochrane Risk of Bias (RoB) tool (The Cochrane Collaboration) was used to evaluate the quality of RCTs. There were a total of 7 evaluation dimensions: random sequence generation; allocation concealment; blinding of participants and personnel; blinding of outcome assessment; incomplete outcome data; selective reporting; and other potential sources of bias. Any discrepancies in the assessments were resolved through a consensus discussion.

### 2.4. Statistical analysis

RevMan 5.4 (The Cochrane Collaboration) and Stata 17.0 (StataCorp LLC) software were used for all meta-analytic analyses. Heterogeneity was quantified using the *I*^2^ statistic. In accordance with common practice in meta-analyses, a fixed-effect model was applied when *I*^2^ was <50%, indicating low heterogeneity; a random-effects model was used when *I*^2^ was 50% or greater, indicating substantial heterogeneity. To assess the robustness of our findings, we additionally performed sensitivity analyses using random-effects models for all outcomes regardless of *I*^2^ values; the results were materially unchanged, confirming that our model selection based on *I*^2^ thresholds did not bias the conclusions. The effect sizes for dichotomous outcomes were expressed as relative risk (RR) with corresponding 95% confidence intervals (CIs). Statistical significance was set at *P* < .05. We assessed the potential for publication bias through visual inspection of funnel plots and performed a sensitivity analysis by sequentially excluding each study to verify the robustness of our meta-analysis results.

## 3. Results

### 3.1. Basic information on study retrieval and selection

A total of 3085 publications were retrieved from the 4 databases. Following the application of the predefined inclusion and exclusion criteria, 28 articles^[11–38]^ were ultimately included in the meta-analysis, comprising 11 RCTs and 17 cohort studies, with a total of 584,249 enrolled patients. The literature screening and inclusion process is shown in Figure 1, and all the baseline characteristics of the included studies are summarized in Table 1.

**Table 1:** Baseline characteristics of included studies.

Author, year	Country	Type of trial	Type of ACS	Follow-up duration	Group	Number of patients (n)	Age (mean ± SD)	Male (%)	Pre MI (%)	Primary outcome
Stone et al, 2006^[11]^	USA	RCT	NSTEMI, UA	30 d	Bivalirudin	9216	62.98 ± 9.83	6413 (69.6)	NA	All-cause mortality, major bleeding, NACE, and MI.
					Heparin	4603	62.95 ± 9.61	3249 (70.6)	NA	
Matar et al, 2006^[12]^	USA	Cohort study	NSTEMI, UA	30 d	Bivalirudin	304	68 ± 11	184 (60.5)	103 (33.9)	Cardiovascular mortality, major bleeding, MACEs, and MI.
					Heparin	283	66 ± 12	166 (58.7)	78 (27.6)	
Stone et al, 2008^[13]^	USA	RCT	STEMI	30 d/1 yr/3 yr	Bivalirudin	1800	59.8 ± 9.8	1388 (77.1)	187/1799 (10.4)	All-cause mortality, cardiovascular mortality, major bleeding, MACEs, NACE, MI, ST, and stroke.
					Heparin	1802	60.6 ± 10.3	1372 (76.1)	205/1800 (11.4)	
Bonello et al, 2009^[14]^	USA	Cohort study	STEMI	In-hospital	Bivalirudin	566	63 ± 14	386 (68.4)	NA	All-cause mortality, cardiovascular mortality, major bleeding, MACEs, ST, and stroke.
					Heparin	333	62.1 ± 13.1	206 (61.9)	NA	
Cortese et al, 2009^[15]^	Italy	Cohort study	UA	In-hospital/30 d	Bivalirudin	50	70 ± 9.2	37 (74)	4 (8)	All-cause mortality, major bleeding, MACEs, MI, and ST.
					Heparin	59	69 ± 9.4	41 (69)	5 (8)	
Feldman et al, 2010^[16]^	USA	Cohort study	NSTEMI	In-hospital	Bivalirudin	461	68.4 ± 12.2	304 (65.9)	178 (38.6)	All-cause mortality, major bleeding, MACEs, MI, and ST.
					Heparin	519	64.5 ± 11.3	383 (73.8)	255 (49.1)	
Lopes et al, 2010^[17]^	USA	Cohort study	STEMI, NSTEMI	In-hospital	Bivalirudin	2634	65.0 ± 14.8	1799 (68.3)	650 (24.7)	All-cause mortality, major bleeding, MI, and stroke.
					Heparin	8451	61.9 ± 14.1	5792 (68.5)	1941 (23)	
Kastrati et al, 2011^[18]^	Germany	RCT	NSTEMI	30 d	Bivalirudin	860	67.5 ± 10.8	661 (76.9)	163 (19.0)	All-cause mortality, major bleeding, MI, ST, and stroke.
					Heparin	861	67.5 ± 11.2	661 (76.8)	188 (21.8)	
Hirschl et al, 2012^[19]^	Austria	Cohort study	STEMI	30 d	Bivalirudin	198	64.6 ± 11.2	129 (65)	22 (11)	All-cause mortality, cardiovascular mortality, major bleeding, MACEs, MI, and stroke.
					Heparin	171	65.5 ± 12.5	115 (67)	17 (10)	
Steg et al, 2013^[22]^	France	RCT	STEMI	30 d/1 yr	Bivalirudin	1089	61.4 ± 14.1	814 (74.7)	80 (7.4)	All-cause mortality, cardiovascular mortality, major bleeding, MACEs, NACE, MI, ST, and stroke.
					Heparin	1109	62.0 + 14.8	861 (77.6)	113 (10.2)	
Sardi et al, 2013^[20]^	USA	Cohort study	STEMI	In-hospital	Bivalirudin	47	56.6 ± 14.79	39 (83)	5 (11.1)	All-cause mortality, major bleeding, MACEs, NACE, MI, and stroke.
					Heparin	57	55.4 ± 10.83	49 (86)	7 (12.7)	
Shelton et al, 2013^[21]^	UK	Cohort study	STEMI	30 d	Bivalirudin	882	63.8 ± 3.1	632 (71.7)	102 (11.6)	All-cause mortality, cardiovascular mortality, major bleeding, MACEs, MI, ST, and stroke.
					Heparin	85	59.2 ± 4.1	63 (74.1)	24 (28.2)	
Sciahbasi et al, 2014^[23]^	Italy	Cohort study	STEMI	30 d	Bivalirudin	154	65 ± 14	121 (79)	21 (14)	All-cause mortality, major bleeding, MACEs, NACE, MI, ST, and stroke.
					Heparin	855	63 ± 12	702 (82)	93 (11)	
Shahzad et al, 2014^[24]^	UK	RCT	STEMI	30 d/1 yr	Bivalirudin	905	63.6 ± 15.1	647 (71.5)	122 (14)	All-cause mortality, cardiovascular mortality, major bleeding, MACEs, MI, and ST.
					Heparin	907	63.8 ± 14.7	663 (73.1)	93 (10)	
Han et al, 2015^[25]^	China	RCT	STEMI, NSTEMI	30 d/1 yr	Bivalirudin	735	57.3 ± 11.6	608 (82.7)	32 (4.4)	All-cause mortality, cardiovascular mortality, major bleeding, MACEs, NACE, MI, ST, and stroke.
					Heparin	1459	58.2 ± 11.8	1194 (81.8)	66 (4.5)	
Valgimigli et al, 2015^[26]^	Switzerland	RCT	STEMI, NSTEMI	30 d/1 yr	Bivalirudin	3610	65.4 ± 11.9	2731 (75.7)	530 (14.7)	All-cause mortality, cardiovascular mortality, major bleeding, MACEs, MI, ST, and stroke.
					Heparin	3603	65.4 ± 11.9	2764 (76.7)	501 (13.9)	
Cascón-Pérez et al, 2016^[27]^	Spain	Cohort study	STEMI, NSTEACS	30 d/6 mo/1 yr	Bivalirudin	582	65.3 ± 12.8	434 (74.6)	NA	All-cause mortality, major bleeding, MI, ST, and stroke.
					Heparin	586	66.5 ± 12	461 (78.7)	NA	
Secemsky et al, 2016^[28]^	USA	Cohort study	STEMI	In-hospital	Bivalirudin	242,087	61.4 ± 12.6	172,622 (71.3)	NA	All-cause mortality, major bleeding, and ST.
					Heparin	271,688	60.7 ± 13.3	195,344 (71.9)	NA	
Erlinge et al, 2017^[31]^	Sweden	RCT	STEMI, NSTEMI	30 d/6 mo/1 yr/2 yr	Bivalirudin	3004	67.3 ± 11.9	2229 (74.2)	490 (16.3)	All-cause mortality, cardiovascular mortality, major bleeding, MACEs, MI, ST, and stroke.
					Heparin	3002	67.6 ± 11.1	2177 (72.5)	484 (16.1)	
Fach et al, 2017^[29]^	Germany	Cohort study	STEMI	In-hospital/1 yr	Bivalirudin	714	63.4 ± 13.4	520 (72.8)	NA	All-cause mortality, major bleeding, MI, and ST.
					Heparin	683	63.8 ± 13.0	514 (75.3)	NA	
Rehmani et al, 2017^[30]^	Australia	Cohort study	ACS	In-hospital	Bivalirudin	1631	62.8 ± 12.2	1281 (78.5)	NA	All-cause mortality, major bleeding, MACEs, and ST.
					Heparin	1740	62.5 ± 12.1	1361 (78.2)	NA	
Zhang et al, 2020^[32]^	China	Cohort study	ACS	30 d	Bivalirudin	361	69.4 ± 10.1	213 (59.0)	89 (24.7)	All-cause mortality, cardiovascular mortality, major bleeding, MACEs, NACE, MI, ST, and stroke.
					Heparin	462	66.4 ± 11.0	252 (54.5)	107 (23.2)	
Li et al, 2022^[34]^	China	RCT	STEMI	30 d	Bivalirudin	3009	60.5 ± 12.1	2350 (78.1)	188 (6.2)	All-cause mortality, cardiovascular mortality, major bleeding, MACEs, NACE, MI, ST, and stroke.
					Heparin	3007	60.6 ± 12.2	2372 (78.9)	197 (6.6)	
Wang et al, 2022^[35]^	China	RCT	STEMI	30 d	Bivalirudin	195	60.27 ± 10.371	79 (75.2)	NA	All-cause mortality, MACEs, MI, and stroke.
					Heparin	105	58.84 ± 10.632	84 (80.0)	NA	
Yu et al, 2022^[33]^	China	Cohort study	STEMI	30 d	Bivalirudin	456	66.7 ± 12.7	326 (71.5)	16 (3.5)	Cardiovascular mortality, major bleeding, MACEs, NACE, MI, ST, and stroke.
					Heparin	344	62.0 ± 13.7	270 (78.5)	28 (8.1)	
Bai et al, 2023^[36]^	China	Cohort study	STEMI	30 d	Bivalirudin	424	63.3 ± 11.3	316 (74.5)	NA	All-cause mortality, cardiovascular mortality, major bleeding, MACEs, NACE, MI, ST, and stroke.
					Heparin	639	64.4 ± 12.0	460 (72.0)	NA	
Hou et al, 2024^[37]^	China	Cohort study	ACS	30 d/6 mo	Bivalirudin	357	63.3 ± 14.8	206 (57.7)	NA	Cardiovascular mortality, major bleeding, MI, and stroke.
					Heparin	385	61.8 ± 14.8	228 (59.2)	NA	
Wu et al, 2025^[38]^	China	RCT	STEMI, NSTEMI	30 d	Bivalirudin	60	58.23 ± 11.17	31 (51.7)	NA	Major bleeding, MACEs, ST, and stroke.
					Heparin	60	59.14 ± 10.46	33 (55.0)	NA	

ACS = acute coronary syndrome, MACEs = major adverse cardiovascular events, MI = myocardial infarction, NA = not available, NACE = net adverse clinical events, NSTEACS = non-ST-elevation acute coronary syndrome, NSTEMI = non-ST-elevation myocardial infarction, RCT = randomized controlled trial, SD = standard deviation, ST = stent thrombosis, STEMI = ST-elevation myocardial infarction, UA = unstable angina.

**Figure 1.**
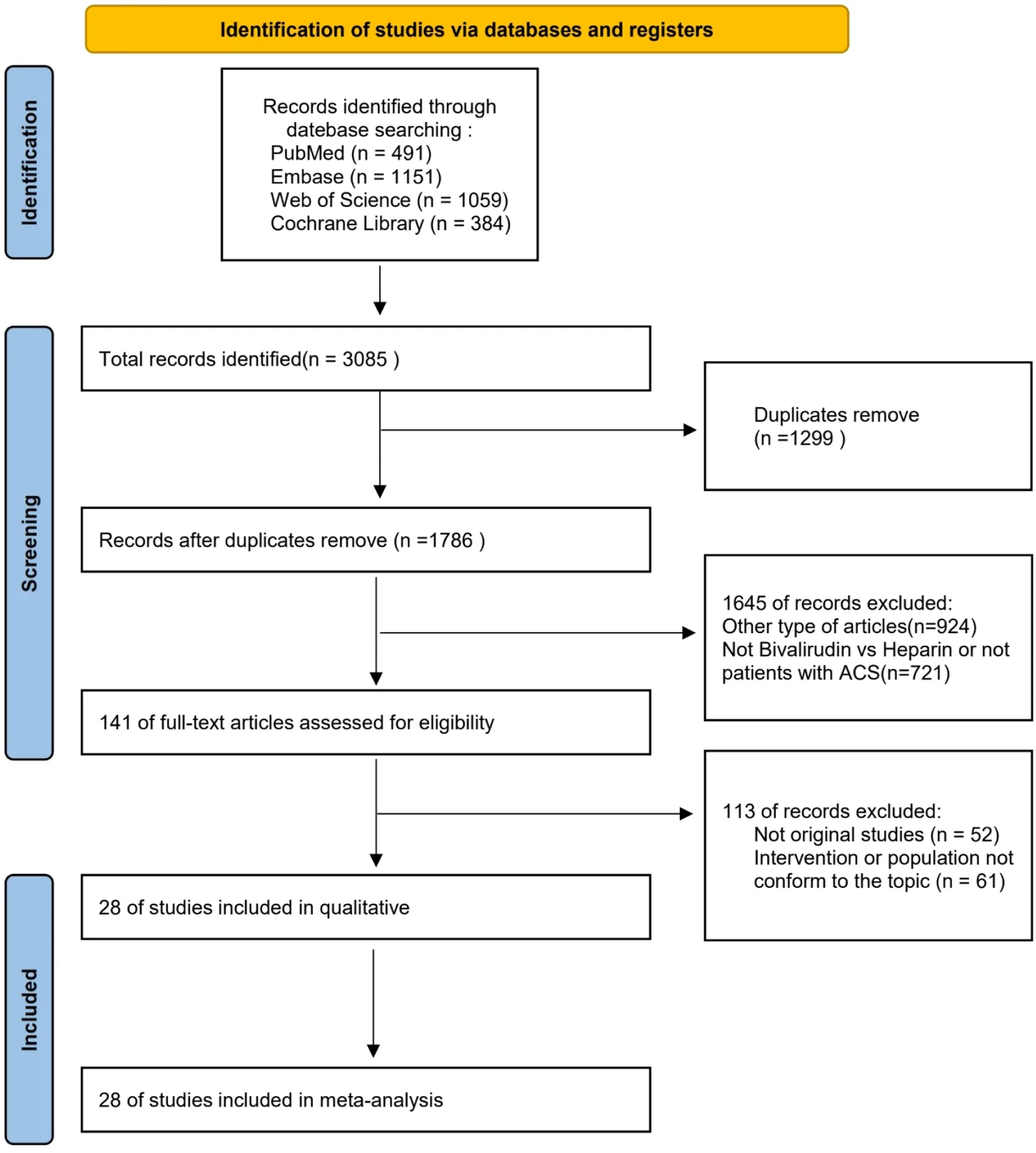
Flowchart of literature search strategies. ACS = acute coronary syndrome.

### 3.2. Quality evaluation

The quality of the included studies was assessed using the RoB (for RCTs) and NOS (for cohort studies). Among the 17 cohort studies included, 5 received a rating of 8 and 12 received a grade of 7, indicating that all the included studies were of good quality. Table 2 presents the specifics of the quality assessment using the NOS. Among the 11 RCTs included, 8 studies demonstrated a low risk of bias in the domain of random sequence generation, and 11 studies demonstrated a low risk of bias in allocation concealment. Conversely, all included trials were classified as having a high risk of bias in terms of blinding of participants and personnel. This is primarily due to the intrinsic connection between perioperative medication interventions for PCI and clinical procedures, which renders participant and investigator blinding infeasible, a common methodological constraint in PCI-related clinical research. For the other bias domains, outcome assessment was blinded in 9 trials, and 10 trials reported complete outcome data. No selective reporting was identified across all 11 trials, and no other potential biases were detected in any of the included studies. A detailed summary of the risk of bias assessment for all RCTs is shown in Figure 2.

**Table 2:** Quality assessment of included cohort studies by Newcastle–Ottawa Scale.

Results of quality assessment using the Newcastle–Ottawa Scale for cohort studies									
Study	Selection				Comparability of cohorts on the basis of the design or study	Outcome			Score
	Representativeness of the exposed cohort	Selection of the nonexposed cohort	Ascertainment of exposure	Demonstration that outcome of interest was not present at start of study		Assessment of outcome	Was follow-up long enough for outcomes to occur	Adequacy of follow-up of cohorts	
Matar et al^[12]^	☆	☆	☆	☆	☆	☆	☆	☆	8
Bonello et al^[14]^	☆	☆	☆	☆	☆	☆	☆		7
Cortese et al^[15]^	☆	☆	☆	☆	☆	☆	☆		7
Feldman et al^[16]^	☆	☆	☆	☆	☆	☆	☆		7
Lopes et al^[17]^	☆	☆	☆	☆	☆	☆	☆		7
Hirschl et al^[19]^	☆		☆	☆	☆	☆	☆	☆	7
Sardi et al^[20]^	☆	☆	☆	☆	☆	☆	☆		7
Shelton et al^[21]^	☆	☆	☆	☆	☆	☆	☆	☆	8
Sciahbasi et al^[23]^	☆	☆	☆	☆	☆	☆	☆		7
Cascón-Pérez et al^[27]^	☆	☆	☆	☆	☆	☆	☆	☆	8
Secemsky et al^[28]^	☆	☆	☆	☆	☆	☆	☆		7
Fach et al^[29]^	☆		☆	☆	☆	☆	☆	☆	7
Rehmani et al^[30]^	☆	☆	☆	☆	☆	☆	☆		7
Zhang et al^[32]^	☆	☆	☆	☆	☆	☆	☆	☆	8
Yu et al^[33]^	☆		☆	☆	☆	☆	☆	☆	7
Bai et al^[36]^	☆	☆	☆	☆	☆	☆	☆		7
Hou et al^[37]^	☆	☆	☆	☆	☆	☆	☆	☆	8

Each “☆” indicates that the corresponding item of the Newcastle-Ottawa Scale was satisfied and represents 1 point; the total score is the sum of all “☆” symbols.

**Figure 2.**
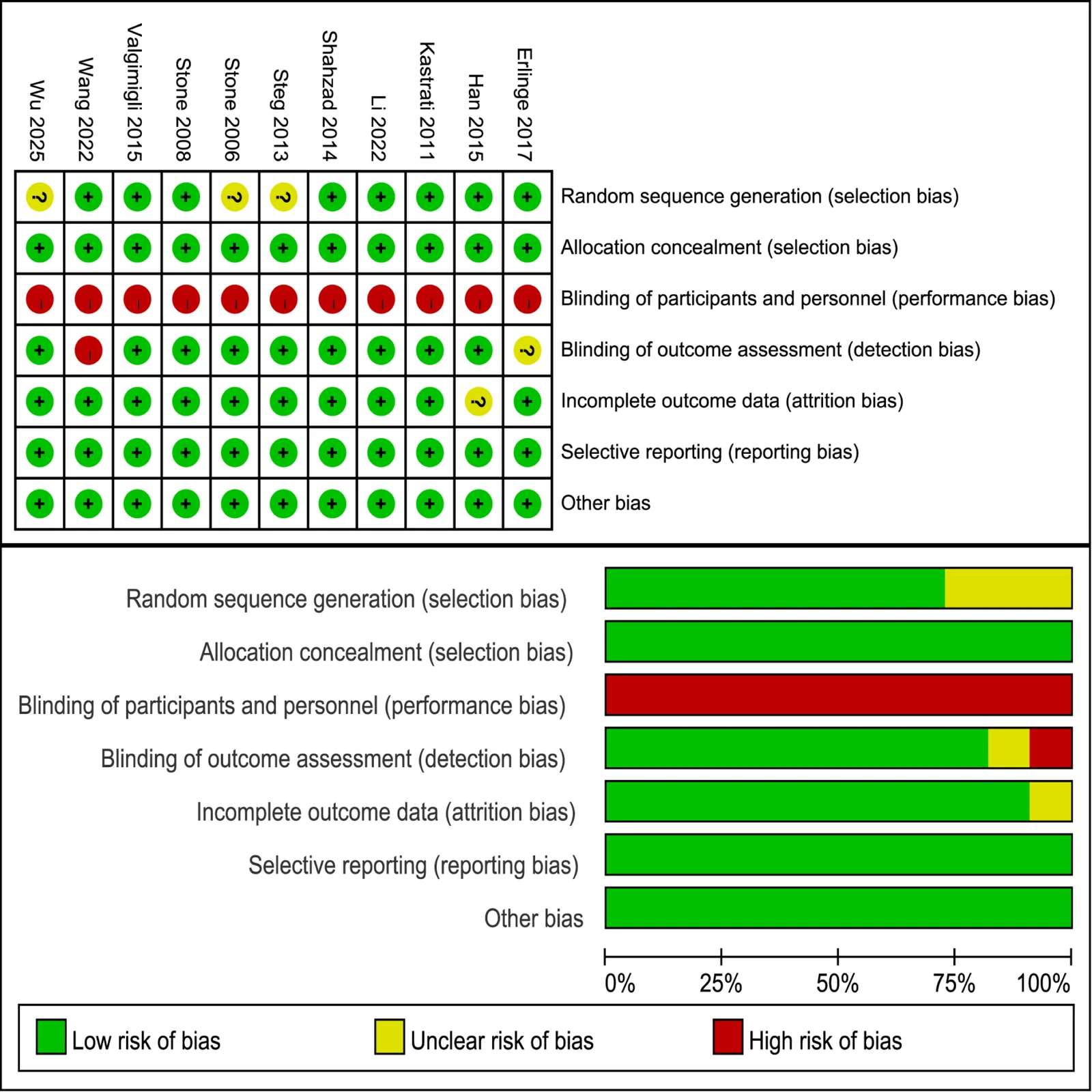
Risk of bias assessment for the included randomized controlled trials.

### 3.3. Clinical outcomes

Table 3 summarizes the meta-analysis results for in‑hospital outcomes, and Table 4 summarizes those for 30‑day outcomes.

**Table 3:** In-hospital outcomes of the meta-analysis.

In-hospital outcomes	No. of studies	Sample size		Heterogeneity		Overall effect size	95% CI of overall effect	*P* value
		Bivalirudin	Heparin	*I*^2^ (%)	*P* value			
All-cause mortality	7	248,140	283,471	0	.78	RR = 0.86	0.84–0.88	<.001
Major bleeding	7	246,559	281,790	51	.06	RR = 0.74	0.62–0.88	<.001
MACEs	3	2658	2592	0	.39	RR = 1.23	0.89–1.68	.21
MI	5	3906	9769	13	.33	RR = 1.24	0.94–1.62	.13
ST	6	245,509	275,022	63	.03	RR = 1.21	0.55–2.67	.63
Stroke	3	3247	8841	0	.94	RR = 0.48	0.20–1.16	.10

CI = confidence interval, MACEs = major adverse cardiovascular events, MI = myocardial infarction, RR = relative risk, ST = stent thrombosis.

**Table 4:** The 30-day outcomes of the meta-analysis.

30-d outcomes	No. of studies	Sample size		Heterogeneity		Overall effect size	95% CI of overall effect	*P* value
		Bivalirudin	Heparin	*I*^2^ (%)	*P* value			
All-cause mortality	17	27,074	23,315	24	.17	RR = 0.85	0.76–0.95	.006
Cardiovascular mortality	13	16,229	16,351	1	.43	RR = 0.81	0.70–0.94	.004
Major bleeding	18	27,946	24,163	67	<.001	RR = 0.59	0.49–0.72	<.001
MACEs	19	27,312	23,416	55	.002	RR = 0.94	0.84–1.06	.31
NACE	9	17,244	14,280	55	.02	RR = 0.79	0.70–0.89	<.001
MI	19	28,141	24,268	27	.14	RR = 0.99	0.90–1.08	.74
ST	15	17,494	18,307	67	<.001	RR = 1.06	0.67–1.69	.80
Stroke	18	18,681	19,442	0	.93	RR = 0.91	0.71–1.16	.44

CI = confidence interval, MACEs = major adverse cardiovascular events, MI = myocardial infarction, NACE = net adverse clinical events, RR = relative risk, ST = stent thrombosis.

#### 3.3.1. In-hospital all-cause mortality

The data were provided in 7^[14,16,17,20,28–30]^ of the 28 articles included. The bivalirudin group had a significantly lower risk of in-hospital all-cause mortality than the heparin group (RR = 0.86, 95% CI: 0.84–0.88, *P* < .001, *I*^2^ = 0%; Fig. S1, Supplemental Digital Content 2). Sensitivity analysis showed that the outcome was stable as a whole (Fig. S2, Supplemental Digital Content 3). The funnel plot did not show significant evidence of publication bias (Fig. S3, Supplemental Digital Content 4).

#### 3.3.2. In-hospital cardiovascular mortality

As only one group of outcome indicators was available for in-hospital cardiovascular mortality, no analysis was performed.

#### 3.3.3. In-hospital major bleeding

The data were provided in 7^[14–17,20,28,29]^ of the 28 articles included. The bivalirudin group had a significantly lower risk of in-hospital major bleeding than the heparin group (RR = 0.74, 95% CI: 0.62–0.88, *P* < .001, *I*^2^ = 51%; Fig. S4, Supplemental Digital Content 5). Sensitivity analysis showed that the outcomes were stable (Fig. S5, Supplemental Digital Content 6). The funnel plot did not show significant evidence of publication bias (Fig. S6, Supplemental Digital Content 7).

#### 3.3.4. In-hospital MACEs

The data were provided in 3^[14,16,30]^ of the 28 articles included. There was no significant difference in the incidence of in-hospital MACEs between the bivalirudin and heparin groups (RR = 1.23, 95% CI: 0.89–1.68, *P* = .21, *I*^2^ = 0%; Fig. S7, Supplemental Digital Content 8). Sensitivity analysis showed that the outcomes were stable (Fig. S8, Supplemental Digital Content 9). The funnel plot did not show significant evidence of publication bias (Fig. S9, Supplemental Digital Content 10).

#### 3.3.5. In-hospital NACE

As only one group of outcome indicators was available for in-hospital NACE, no analysis was performed.

#### 3.3.6. In-hospital MI

The data were provided in 5^[15–17,20,29]^ of the 28 articles included. There was no significant difference in the incidence of in-hospital MI between the bivalirudin and heparin groups (RR = 1.24, 95% CI: 0.94–1.62, *P* = .13, *I*^2^ = 13%; Fig. S10, Supplemental Digital Content 11). Sensitivity analysis showed that the outcomes were stable (Fig. S11, Supplemental Digital Content 12). The funnel plot did not show significant evidence of publication bias (Fig. S12, Supplemental Digital Content 13).

#### 3.3.7. In-hospital ST

The data were provided in 6^[14–16,28–30]^ of the 28 articles included. There was no significant difference in the incidence of in-hospital ST between the bivalirudin and heparin groups (RR = 1.21, 95% CI: 0.55–2.67, *P* = .63, *I*^2^ = 63%; Fig. S13, Supplemental Digital Content 14). Sensitivity analysis showed that the outcomes were unstable (Fig. S14, Supplemental Digital Content 15). The funnel plot did not show significant evidence of publication bias (Fig. S15, Supplemental Digital Content 16).

#### 3.3.8. In-hospital stroke

The data were provided in 3^[14,17,20]^ of the 28 articles included. There was no significant difference in the incidence of in-hospital stroke between the bivalirudin and heparin groups (RR = 0.48, 95% CI: 0.20–1.16, *P* = .10, *I*^2^ = 0%; Fig. S16, Supplemental Digital Content 17). Sensitivity analysis showed that the outcomes were stable (Fig. S17, Supplemental Digital Content 18). The funnel plot did not show significant evidence of publication bias (Fig. S18, Supplemental Digital Content 19).

#### 3.3.9. 30-day all-cause mortality

The data were provided in 17^[11,13,15,18,19,21–27,31,32,34–36]^ of the 28 articles included. The bivalirudin group had a significantly lower risk of 30-day all-cause mortality than the heparin group (RR = 0.85, 95% CI: 0.76–0.95, *P* = .006, *I*^2^ = 24%; Fig. S19, Supplemental Digital Content 20). Sensitivity analysis showed that the outcomes were stable (Fig. S20, Supplemental Digital Content 21). The funnel plot did not show significant evidence of publication bias (Fig. S21, Supplemental Digital Content 22).

#### 3.3.10. 30-day cardiovascular mortality

The data were provided in 13^[12,13,19,21,22,25,26,31–34,36,37]^ of the 28 articles included. The bivalirudin group had a significantly lower risk of 30-day cardiovascular mortality than the heparin group (RR = 0.81, 95% CI: 0.70–0.94, *P* = .004, *I*^2^ = 1%; Fig. S22, Supplemental Digital Content 23). Sensitivity analysis showed that the outcomes were stable (Fig. S23, Supplemental Digital Content 24). The funnel plot did not show significant evidence of publication bias (Fig. S24, Supplemental Digital Content 25).

#### 3.3.11. 30-day major bleeding

The data were provided in 18^[11–13,18,19,21–27,31–34,36,37]^ of the 28 articles included. The bivalirudin group had a significantly lower risk of 30-day major bleeding than the heparin group (RR = 0.59, 95% CI: 0.49–0.72, *P* < .001, *I*^2^ = 67%; Fig. S25, Supplemental Digital Content 26). Sensitivity analysis showed that the outcomes were stable (Fig. S26, Supplemental Digital Content 27). The funnel plot did not show significant evidence of publication bias (Fig. S27, Supplemental Digital Content 28).

#### 3.3.12. 30-day MACEs

The data were provided in 19^[11–13,15,18,19,21–26,31–36,38]^ of the 28 articles included. There was no significant difference in the incidence of 30-day MACEs between the bivalirudin and heparin groups (RR = 0.94, 95% CI: 0.84–1.06, *P* = .31, *I*^2^ = 55%; Fig. S28, Supplemental Digital Content 29). Sensitivity analysis showed that the outcomes were stable (Fig. S29, Supplemental Digital Content 30). The funnel plot did not show significant evidence of publication bias (Fig. S30, Supplemental Digital Content 31).

#### 3.3.13. 30-day NACE

The data were provided in 9^[11,13,22,23,25,32–34,36]^ of the 28 articles included. The bivalirudin group had a significantly lower risk of 30-day NACE than the heparin group (RR = 0.79, 95% CI: 0.70–0.89, *P* < .001, *I*^2^ = 55%; Fig. S31, Supplemental Digital Content 32). Sensitivity analysis showed that the outcomes were stable (Fig. S32, Supplemental Digital Content 33). The funnel plot did not show significant evidence of publication bias (Fig. S33, Supplemental Digital Content 34).

#### 3.3.14. 30-day MI

The data were provided in 19^[11–13,18,19,21–27,31–37]^ of the 28 articles included. There was no significant difference in the incidence of 30-day MI between the bivalirudin and heparin groups (RR = 0.99, 95% CI: 0.90–1.08, *P* = .74, *I*^2^ = 27%; Fig. S34, Supplemental Digital Content 35). Sensitivity analysis showed that the outcomes were stable (Fig. S35, Supplemental Digital Content 36). The funnel plot did not show significant evidence of publication bias (Fig. S36, Supplemental Digital Content 37).

#### 3.3.15. 30-day ST

The data were provided in 15^[13,18,21–27,31–34,36,38]^ of the 28 articles included. There was no significant difference in the incidence of 30-day ST between the bivalirudin and heparin groups (RR = 1.06, 95% CI: 0.67–1.69, *P* = .80, *I*^2^ = 67%; Fig. S37, Supplemental Digital Content 38). Sensitivity analysis showed that the outcomes were stable (Fig. S38, Supplemental Digital Content 39). The funnel plot did not show significant evidence of publication bias (Fig. S39, Supplemental Digital Content 40).

#### 3.3.16. 30-day stroke

The data were provided in 18^[13,18,19,21–27,31–38]^ of the 28 articles included. There was no significant difference in the incidence of 30-day stroke between the bivalirudin and heparin groups (RR = 0.91, 95% CI: 0.71–1.16, *P* = .44, *I*^2^ = 0%; Fig. S40, Supplemental Digital Content 41). Sensitivity analysis showed that the outcomes were stable (Fig. S41, Supplemental Digital Content 42). The funnel plot did not show significant evidence of publication bias (Fig. S42, Supplemental Digital Content 43).

### 3.4. Subgroup analysis

Based on the 11 RCTs and 17 cohort studies, we performed separate meta-analyses for 30-day outcomes. Table 5 presents the meta‑analysis results for the 11 RCTs, and Table 6 presents the corresponding results for the 17 cohort studies.

**Table 5:** Subgroup analysis of randomized controlled trials in 30-day outcomes.

30-d outcomes of randomized controlled trials	No. of studies	Sample size		Heterogeneity		Overall effect size	95% CI of overall effect	*P* value
		Bivalirudin	Heparin	*I*^2^ (%)	*P* value			
All-cause mortality	10	24,423	20,458	30	.17	RR = 0.90	0.79–1.02	.11
Cardiovascular mortality	6	13,247	13,982	0	.44	RR = 0.79	0.67–0.92	.003
Major bleeding	9	24,228	20,353	77	<.001	RR = 0.65	0.52–0.81	<.001
MACEs	11	24,483	20,518	55	.01	RR = 1.01	0.90–1.14	.81
NACE	5	15,849	11,980	57	.06	RR = 0.84	0.74–0.97	.01
MI	10	24,423	20,458	43	.08	RR = 1.03	0.93–1.13	.59
ST	9	14,635	15,336	78	<.001	RR = 1.11	0.60–2.05	.74
Stroke	10	15,267	15,915	0	.88	RR = 0.98	0.74–1.30	.89

CI = confidence interval, MACEs = major adverse cardiovascular events, MI = myocardial infarction, NACE = net adverse clinical events, RR = relative risk, ST = stent thrombosis.

**Table 6:** Subgroup analysis of cohort studies in 30-day outcomes.

30-d outcomes of cohort studies	No. of studies	Sample size		Heterogeneity		Overall effect size	95% CI of overall effect	*P* value
		Bivalirudin	Heparin	*I*^2^ (%)	*P* value			
All-cause mortality	7	2651	2857	0	.66	RR = 0.65	0.50–0.86	.002
Cardiovascular mortality	7	2982	2369	12	.34	RR = 0.94	0.67–1.31	.71
Major bleeding	9	3718	3810	0	.52	RR = 0.46	0.34–0.63	<.001
MACEs	8	2829	2898	21	.26	RR = 0.74	0.61–0.90	.002
NACE	4	1395	2300	0	.63	RR = 0.67	0.56–0.81	<.001
MI	9	3718	3810	0	.93	RR = 0.69	0.51–0.91	.01
ST	6	2859	2971	22	.26	RR = 1.00	0.60–1.69	.99
Stroke	8	3414	3527	0	.81	RR = 0.70	0.42–1.18	.19

CI = confidence interval, MACEs = major adverse cardiovascular events, MI = myocardial infarction, NACE = net adverse clinical events, RR = relative risk, ST = stent thrombosis.

For RCTs, bivalirudin significantly reduced cardiovascular mortality (RR = 0.79, 95% CI: 0.67–0.92, *P* = .003, *I*^2^ = 0%), major bleeding (RR = 0.65, 95% CI: 0.52–0.81, *P* < .001, *I*^2^ = 77%), and NACE (RR = 0.84, 95% CI: 0.74–0.97, *P* = .01, *I*^2^ = 57%), but not all-cause mortality (RR = 0.90, 95% CI: 0.79–1.02, *P* = .11, *I*^2^ = 30%). No significant differences were observed in MACEs (RR = 1.01, 95% CI: 0.90–1.14, *P* = .81, *I*^2^ = 55%), MI (RR = 1.03, 95% CI: 0.93–1.13, *P* = .59, *I*^2^ = 43%), ST (RR = 1.11, 95% CI: 0.60–2.05, *P* = .74, *I*^2^ = 78%), or stroke (RR = 0.98, 95% CI: 0.74–1.30, *P* = .89, *I*^2^ = 0%; Figs. S43, S44, S45, S46, S47, S48, S49 and S50, Supplemental Digital Content 44).

For cohort studies, bivalirudin was associated with significantly lower all-cause mortality (RR = 0.65, 95% CI: 0.50–0.86, *P* = .002, *I*^2^ = 0%), major bleeding (RR = 0.46, 95% CI: 0.34–0.63, *P* < .001, *I*^2^ = 0%), MACEs (RR = 0.74, 95% CI: 0.61–0.90, *P* = .002, *I*^2^ = 21%), NACE (RR = 0.67, 95% CI: 0.56–0.81, *P* < .001, *I*^2^ = 0%), and MI (RR = 0.69, 95% CI: 0.51–0.91, *P* = .01, *I*^2^ = 0%). No significant differences were observed in cardiovascular mortality (RR = 0.94, 95% CI: 0.67–1.31, *P* = .71, *I*^2^ = 12%), ST (RR = 1.00, 95% CI: 0.60–1.69, *P* = .99, *I*^2^ = 22%), and stroke (RR = 0.77, 95% CI: 0.42–1.18, *P* = .19, *I*^2^ = 0%; Figs. S51, S52, S53, S54, S55, S56, S57 and S58, Supplemental Digital Content 45).

## 4. Discussion

### 4.1. General interpretation of the results in the context of other evidence

Coronary artery disease remains a leading cause of global mortality and morbidity, and ACS constitutes a substantial proportion of this disease burden, leading to millions of hospitalizations and deaths worldwide each year.^[39]^ PCI has emerged as a cornerstone in the management of ACS, with anticoagulation acting as a critical adjunctive therapy to prevent periprocedural thrombosis and optimize clinical outcomes.^[40]^ The selection of anticoagulant therapy directly modulates the delicate balance between ischemic and bleeding risks – a long-standing clinical dilemma, particularly among ACS patients, who are at high risk of mortality from both recurrent ischemia and major bleeding events.^[2]^

As previously highlighted, a prior meta-analysis^[3]^ demonstrated that bivalirudin, compared with unfractionated heparin (UFH), reduces major bleeding and cardiovascular mortality in MI patients undergoing PCI, without significant differences in MACEs, all-cause mortality, recurrent MI, stroke, or ST. However, these conclusions are limited by several critical shortcomings: the inclusion of only 10 trials restricts statistical power for infrequent outcomes (increasing the risk of type II error); potential publication bias may overestimate treatment benefits; and scarce safety endpoints cannot be robustly evaluated.^[41]^ In contrast, the present meta-analysis overcomes these limitations and improves clinical relevance by integrating 28 eligible studies (comprising 584,249 patients) and targeting the broader ACS population, rather than the narrower MI cohort. Methodologically, the expanded sample size substantially enhances statistical power, permitting conclusive evaluation of infrequent outcomes and reducing type II error risk. It also includes more studies to mitigate publication bias.^[42,43]^ From a clinical perspective, the ACS-focused design offers distinct advantages: by encompassing STEMI, NSTEMI, and UA, it closely reflects real-world clinical practice, thereby enhancing the external validity and generalizability of the study findings. Furthermore, this design aligns with current clinical guidelines that advocate standardized antithrombotic management across all ACS presentations, making the conclusions highly relevant to contemporary clinical decision-making.^[44]^ Our meta-analysis delivers robust evidence that bivalirudin outperforms heparin in ACS patients undergoing PCI across critical safety and composite endpoints. Specifically, bivalirudin was associated with significant reductions in in-hospital all-cause mortality and major bleeding, with sustained benefits evident at 30 days – including lower cardiovascular mortality, major bleeding events, and NACE. Importantly, these advantages were achieved without compromising ischemic outcomes, as no significant differences were observed in MACEs, MI, ST, or stroke between the 2 groups at either evaluation point. The observed reductions in major bleeding and mortality are clinically relevant because even moderate RRs translate into meaningful absolute benefits given the high baseline event rates in this population, and these findings are consistent with prior meta-analyses.

The superiority of bivalirudin over heparin in terms of key safety and composite endpoints among ACS patients undergoing PCI stems from fundamental differences in their anticoagulant mechanisms, pharmacokinetics, as well as action specificity, which are highly compatible with the pathophysiological characteristics of ACS.^[45]^ As a direct thrombin inhibitor, bivalirudin binds reversibly to both the active site and substrate-binding site of thrombin without relying on antithrombin III, exerting potent inhibitory effects on both free thrombin and fibrin-bound thrombin – the latter being a critical driver of thrombus formation and in-ST recurrence in ACS.^[46]^ It also exhibits a linear dose-response relationship with minimal interindividual variability, ensuring stable and controllable anticoagulation efficacy.^[47]^ In contrast, UFH acts as an indirect anticoagulant whose efficacy is heavily dependent on antithrombin III levels, resulting in substantial variability; moreover, its inhibitory effect on fibrin-bound thrombin is weak.^[48]^ Additionally, bivalirudin has a short half-life, with anticoagulant effects abating rapidly after discontinuation without the need for a neutralizing agent. Unlike UFH, it does not bind to platelet factor 4 and thus rarely induces heparin-induced thrombocytopenia, nor does it interfere with platelet function.^[49–51]^ This optimal synergy with antiplatelet agents fundamentally reduces the risk of severe bleeding events such as puncture site hematomas and intracranial hemorrhage. Since bleeding is a major independent risk factor for early mortality in post-PCI ACS patients, the reduction in bleeding incidence directly lowers fatal complications, including transfusion-related adverse reactions, bleeding-induced myocardial ischemic reperfusion injury, and multiple organ failure, thereby decreasing in-hospital all-cause mortality. Such short-term benefits persist up to 30 days because bivalirudin achieves comprehensive thrombus inhibition with a lower bleeding risk, avoiding the need to discontinue antiplatelet therapy due to bleeding events and thus maintaining effective ischemic event prevention. This translates to reduced 30-day cardiovascular mortality and NACE. It is worth noting that no significant difference was observed in the ischemic outcomes of the 2 groups, as bivalirudin enables precise control of anticoagulant intensity that can be adjusted based on patient weight and renal function, maintaining effective periprocedural anticoagulation concentrations sufficient to prevent ST, which is comparable to heparin in terms of ischemic prevention efficacy.^[52]^ Consequently, there are no statistically significant differences in MACEs, MI, ST, or stroke between the bivalirudin and heparin groups at either time point.

In addition, a particularly important finding emerged from the subgroup analyses: the reduction in all-cause mortality was statistically significant only in the cohort studies (RR = 0.65, 95% CI: 0.50–0.86, *P* = .002, *I*^2^ = 0%) but not in the RCTs (RR = 0.90, 95% CI: 0.79–1.02, *P* = .11, *I*^2^ = 30%). This discrepancy is crucial for interpreting the overall pooled result (RR = 0.85, 95% CI: 0.76–0.95, *P* = .006, *I*^2^ = 24%), as the significant overall estimate appears to be driven largely by the observational data. The lack of a mortality benefit in the more rigorously controlled RCTs suggests that the mortality advantage observed in the pooled analysis should be interpreted with caution. Potential explanations for this discordance include residual confounding in observational studies (e.g., unmeasured differences in baseline risk profiles, operator experience, or concomitant therapies), as well as possible selection biases where clinicians may have preferentially assigned lower-risk patients to bivalirudin. This finding underscores that the current evidence does not support a definitive mortality benefit for bivalirudin over heparin and reinforces the need for additional large-scale RCTs with adequate power to detect mortality differences.

### 4.2. Limitations of the evidence included in the review

Firstly, the majority of the included studies were cohort studies, and a relatively limited number of RCTs, which impairs the overall quality and reliability of the findings. Secondly, the sample size of the observed data during hospitalization was limited, and the sample size of some studies might be too small or too large, which reduced the reliability of these results. Thirdly, it is important to note the reliability of the funnel plot for studies with a small sample size because the funnel plot does not show obvious asymmetry, but this does not precisely mean the absence of publication bias. Additionally, among the included studies, although 8 studies reported the results of the 1-year follow-up, the relevant data that can be analyzed are still scarce; at the same time, all other studies did not report long-term safety and efficacy endpoint indicators such as mortality, late ST, and recurrent stroke, precluding any analysis of the drug’s long-term effects. Finally, there are differences in the baseline characteristics between the studies, which may introduce selection bias.

Furthermore, sensitivity analysis showed that the overall result was generally robust, as excluding any single study except Rehmani et al^[30]^ did not alter the null conclusion (all CIs remained crossing 1). Although Cortese et al^[15]^ also had a point estimate favoring bivalirudin (RR = 0.39), its small weight (5.3%) and very low event count (0 vs 1) meant its exclusion had no meaningful impact. In contrast, after removing Rehmani et al^[30]^ – the only study with both a substantial weight (27.9%) and a point estimate favoring bivalirudin (RR = 0.50) – the pooled 95% CI shifted to lying entirely above 1, indicating a statistically significant higher risk of ST with bivalirudin. Thus, the overall null finding is not fully robust and should be interpreted with caution, as it is largely driven by the inclusion of Rehmani et al.^[30]^ Moreover, differences in baseline characteristics and radial artery access in Rehmani et al^[30]^ compared with other large studies may have contributed to its opposing point estimate and the resulting sensitivity.

### 4.3. Limitations of the literature review process

First, despite comprehensive searches across PubMed, Embase, Web of Science, and The Cochrane Library, potential selection bias may exist due to database indexing variations, language restrictions, or unretrieved unpublished literature. Moreover, the discrepancy between RCTs and cohort studies – for example, the reduction in all-cause mortality was statistically significant only in the cohort studies but not in the RCTs – suggests possible residual confounding that may affect the reliability of our conclusions. Furthermore, significant statistical heterogeneity was observed for key outcomes (e.g., major bleeding, NACE), which likely arises from multiple sources, including inconsistent baseline characteristics (ACS subtypes, antiplatelet regimens, comorbidities) and divergent bleeding definitions across trials. Additionally, variations in intraoperative surgical approaches may also impact clinical outcomes. However, due to insufficient standardized individual patient data and inconsistent reporting of stratification factors, we could not perform targeted subgroup analyses or meta-regression to quantitatively explore these sources of heterogeneity, which limits our ability to assess their individual impacts. Finally, the geographical homogeneity of the included studies – with study populations predominantly derived from middle- and high-income countries – limits the external validity and generalizability of the findings to the global cohort of ACS patients undergoing PCI.

### 4.4. Implication of research results for clinical practice, policy formulation, and future research

The findings suggest that bivalirudin may be considered over heparin in ACS patients undergoing PCI, especially those at high bleeding risk – such as those with a history of heparin-induced thrombocytopenia, renal dysfunction, or concurrent use of multiple antiplatelet agents. Clinicians can also leverage its predictable pharmacokinetics and reversible action to simplify perioperative anticoagulation management, given its proven short-term benefits, including reduced in-hospital mortality, major bleeding, and 30-day NACE, all without compromising ischemic outcomes.

In terms of health policy, our analysis suggests that guideline committees may consider updating recommendations to reflect bivalirudin as a reasonable alternative to heparin for ACS patients undergoing PCI, particularly in those at elevated bleeding risk. Additionally, efforts should be made to optimize the accessibility and cost-effectiveness of bivalirudin in regions with a high incidence of ACS, while funding cross-regional clinical registry programs to collect real-world data for validating its efficacy across diverse healthcare settings. However, the feasibility of this approach still requires further validation from more high-quality studies. For future research, priorities should include conducting large-scale, long-term RCTs (with ≥1 year of follow-up) to evaluate bivalirudin’s long-term endpoints, such as late ST and recurrent stroke, in underrepresented populations such as those from low- and middle-income countries; focusing on subgroup analyses to identify optimal candidates, including different ACS subtypes, elderly patients, diabetic patients, and those with renal failure; performing network meta-analyses to compare bivalirudin with other common PCI anticoagulants and combined regimens; and utilizing large real-world databases to bridge the gap between RCT findings and routine clinical practice.

In conclusion, this meta-analysis suggests that bivalirudin may offer advantages over heparin in reducing in-hospital and 30-day mortality and major bleeding in patients with ACS undergoing PCI, with no significant differences in ischemic outcomes such as MACEs, MI, ST, or stroke. These findings suggest that bivalirudin may be a reasonable alternative to heparin in selected patients, but more large-scale, long-term RCTs are needed to confirm these results.

## Acknowledgments

The authors thank all contributors who provided valuable support during the conduct of this study.

## Author contributions

**Data curation:** Yuanming Liao, Yanzi Mai.

**Formal analysis:** Chen Yang, Zhen Ning.

**Writing – original draft:** Chen Yang, Zhen Ning, Weiming Liang, Yuanming Liao, Yanzi Mai.

**Writing – review & editing:** Chen Yang, Zhen Ning, Weiming Liang, Jie Li.




























































































































