# Supplementary material for: Comparison of bivalirudin versus heparin in percutaneous coronary intervention for patients with acute coronary syndrome: A meta-analysis
Source: Medicine (Baltimore). 2026 Sep 25;105(39):e50046. doi: 10.1097/MD.0000000000050046 (PMC13619192; doi:10.1097/MD.0000000000050046)
Supplement: Supplementary file 1 [file medi-105-e50046-s001.docx]

Supplementary Table 1. Search strategies and retrieved results in PubMed database (search date: August 8, 2025)

| PubMed | | |
| --- | --- | --- |
| No. | Query | Results |
| 1 | ("bivalirudin" [Supplementary Concept]) OR (((((((((((bivalirudin[Title/Abstract]) OR (Hirudin[Title/Abstract])) OR (BG 8967[Title/Abstract])) OR (BG-8967[Title/Abstract])) OR (BG8967[Title/Abstract])) OR (Hirulog[Title/Abstract])) OR (Hirulog-1[Title/Abstract])) OR (Angiomax[Title/Abstract])) OR (Angiomax RTU[Title/Abstract])) OR (CTB-001[Title/Abstract])) OR (bivalirudin trifluoroacetate[Title/Abstract])) | 5,084 results |
| 2 | ("Heparin"[Mesh]) OR ((((((((((heparin[Title/Abstract]) OR (Heparinic Acid[Title/Abstract])) OR (Unfractionated Heparin[Title/Abstract])) OR (Heparin, Unfractionated[Title/Abstract])) OR (Sodium Heparin[Title/Abstract])) OR (Heparin, Sodium[Title/Abstract])) OR (Heparin Sodium[Title/Abstract])) OR (alpha-Heparin[Title/Abstract])) OR (alpha Heparin[Title/Abstract])) OR (Liquaemin[Title/Abstract])) | 110,811 results |
| 3 | ("Percutaneous Coronary Intervention"[Mesh]) OR ((((((((((((Percutaneous Coronary Intervention[Title/Abstract]) OR (Coronary Intervention, Percutaneous[Title/Abstract])) OR (Coronary Interventions, Percutaneous[Title/Abstract])) OR (Intervention, Percutaneous Coronary[Title/Abstract])) OR (Interventions, Percutaneous Coronary[Title/Abstract])) OR (Percutaneous Coronary Interventions[Title/Abstract])) OR (Percutaneous Coronary Revascularization[Title/Abstract])) OR (Coronary Revascularization, Percutaneous[Title/Abstract])) OR (Coronary Revascularizations, Percutaneous[Title/Abstract])) OR (Percutaneous Coronary Revascularizations[Title/Abstract])) OR (Revascularization, Percutaneous Coronary[Title/Abstract])) OR (Revascularizations, Percutaneous Coronary[Title/Abstract])) | 88,764 results |
| 4 | (((randomized controlled trial [pt] OR controlled clinical trial [pt] OR randomized [tiab] OR placebo [tiab] OR clinical trials as topic [mh] OR randomly [tiab] OR trial [ti]) NOT (animals [mh] NOT humans [mh])) OR (Prospective[Title/Abstract])) OR (Retrospective[Title/Abstract]) | 3,156,834 results |
| 5 | 1 AND 2 AND 3 | 814 results |
| 6 | 1 AND 2 AND 3 AND 4 | 491 results |

tiab: Title/Abstract; mh: MeSH Heading; pt: Publication Type; ti: Title; Mesh: Medical Subject Headings
