# Supplementary material for: Comparison of bivalirudin versus heparin in percutaneous coronary intervention for patients with acute coronary syndrome: A meta-analysis
Source: Medicine (Baltimore). 2026 Sep 25;105(39):e50046. doi: 10.1097/MD.0000000000050046 (PMC13619192; doi:10.1097/MD.0000000000050046)
Supplement: Supplementary file 2 [file medi-105-e50046-s046.docx]

Supplementary Table 2 Search strategies and retrieved results in Web of Science database (search date: August 8, 2025)

| Web of Science | | |
| --- | --- | --- |
| No. | Query | Results |
| 1 | TS=(bivalirudin OR Hirudin OR BG 8967 OR BG-8967 OR BG8967 OR Hirulog OR Hirulog-1 OR Angiomax OR Angiomax RTU OR CTB-001 OR bivalirudin trifluoroacetate) and Preprint Citation Index (Exclude – Database) | 11,147 results |
| 2 | TS=(heparin OR Heparinic Acid OR Unfractionated Heparin OR Heparin, Unfractionated OR Sodium Heparin OR Heparin, Sodium OR Heparin Sodium OR alpha-Heparin OR alpha Heparin OR Liquaemin) and Preprint Citation Index (Exclude – Database) | 188,055 results |
| 3 | TS=(Percutaneous Coronary Intervention OR Coronary Intervention, Percutaneous OR Coronary Interventions, Percutaneous OR Intervention, Percutaneous Coronary OR Interventions, Percutaneous Coronary OR Percutaneous Coronary Interventions OR Percutaneous Coronary Revascularization OR Coronary Revascularization, Percutaneous OR Coronary Revascularizations, Percutaneous OR Percutaneous Coronary Revascularizations OR Revascularization, Percutaneous Coronary OR Revascularizations, Percutaneous Coronary) and Preprint Citation Index (Exclude – Database) | 116,770  results |
| 4 | ((((((((TS=(randomized controlled trial )) OR TS=(controlled clinical trial )) OR TS=(Randomized)) OR TS=(placebo )) OR TS=(clinical trials as topic )) OR TS=(randomly )) OR TS=(Trial)) OR TS=(Prospective )) OR TS=(Retrospective) | 6,971,091 results |
| 5 | 1 AND 2 AND 3 AND 4 | 1,059 results |

TS: Topic Search
