# Supplementary material for: Comparison of bivalirudin versus heparin in percutaneous coronary intervention for patients with acute coronary syndrome: A meta-analysis
Source: Medicine (Baltimore). 2026 Sep 25;105(39):e50046. doi: 10.1097/MD.0000000000050046 (PMC13619192; doi:10.1097/MD.0000000000050046)
Supplement: Supplementary file 3 [file medi-105-e50046-s047.docx]

Supplementary Table 3 Search strategies and retrieved results in Embase database (search date: August 8, 2025)

| Embase | | |
| --- | --- | --- |
| No. | Query | Results |
| 1 | 'bivalirudin'/exp | 8,244 results |
| 2 | (('angiomax':ab,ti OR 'angiomax rtu':ab,ti OR 'angiox':ab,ti OR 'bg 8967':ab,ti OR 'bg8967':ab,ti OR 'bivalirudin in sodium chloride 0.9%':ab,ti OR 'bivalurudin trifluoroacetate':ab,ti OR 'd phe pro arg pro':ab,ti) AND gly:ab,ti AND '4 desulfohirudin [53-64]':ab,ti OR 'd phe pro arg pro gly gly gly gly asn gly asp phe glu glu ile pro glu glu tyr leu':ab,ti OR 'dextro phenylalanylprolylarginylprolylglycylglycylglycylglycylasparaginylglycyl alpha aspartylphenylalanyl alpha glutamyl alpha glutamylisoleucylprolyl alpha glutamyl alpha glutamyl alpha tyrosyleucine':ab,ti OR 'dextro phenylalanylprolylarginylprolylglycylglycylglycylglycylasparaginylglycyl alpha aspartylphenylalanyl alpha glutamyl alpha glutamylisoleucylprolyl alpha glutamyl alpha glutamyl alpha tyrosyleucine bis':ab,ti) AND '2, 2, 2 trifluoroacetate':ab,ti OR 'ep 6101':ab,ti OR 'ep6101':ab,ti OR 'hirulog':ab,ti OR 'hirulog 1':ab,ti OR 'bivalirudin':ab,ti | 3,535 results |
| 3 | 'heparin'/exp | 257,392 results |
| 4 | ((((('alpha heparin':ab,ti OR 'ammonium heparinate':ab,ti OR 'benzalkonium heparin':ab,ti OR 'beparine':ab,ti OR 'clarin':ab,ti OR 'contusol':ab,ti OR 'disebrin':ab,ti OR 'eleparon':ab,ti OR 'elheparin':ab,ti OR 'elheparon':ab,ti OR 'endogenous heparin':ab,ti OR 'epiheparin':ab,ti OR 'gag 98':ab,ti OR 'helberina':ab,ti OR 'hep flush kit':ab,ti OR 'hep lock':ab,ti OR 'hep-lock':ab,ti OR 'hep-lock u/p':ab,ti OR 'hep-pak cvc':ab,ti OR 'hepaflex':ab,ti OR 'hepalean':ab,ti OR 'heparin injection b.p.':ab,ti OR 'heparin leo':ab,ti OR 'heparin lock flush':ab,ti OR 'heparin lock flush plus sodium chloride':ab,ti OR 'heparin lock flush preservative free':ab,ti OR 'heparin monosulfate':ab,ti OR 'heparin monosulphate':ab,ti OR 'heparin novo':ab,ti OR 'heparin ointment':ab,ti OR 'heparin potassium':ab,ti OR 'heparin sodium':ab,ti OR 'heparin sodium 1, 000 units':ab,ti) AND 'sodium chloride 0.9%':ab,ti OR 'heparin sodium 1, 000 units in dextrose 5%':ab,ti OR 'heparin sodium 1, 000 units in sodium chloride 0.9%':ab,ti OR 'heparin sodium 10, 000 units in dextrose 5%':ab,ti OR 'heparin sodium 10, 000 units in sodium chloride 0.45%':ab,ti OR 'heparin sodium 10, 000 units in sodium chloride 0.9%':ab,ti OR 'heparin sodium 12, 500 units in dextrose 5%':ab,ti OR 'heparin sodium 12, 500 units in sodium chloride 0.45%':ab,ti OR 'heparin sodium 12, 500 units in sodium chloride 0.9%':ab,ti OR 'heparin sodium 2, 000 units':ab,ti) AND 'sodium chloride 0.9%':ab,ti OR 'heparin sodium 2, 000 units in dextrose 5%':ab,ti OR 'heparin sodium 2, 000 units in sodium chloride 0.9%':ab,ti OR 'heparin sodium 20, 000 units':ab,ti) AND 'dextrose 5%':ab,ti OR 'heparin sodium 20, 000 units in dextrose 5%':ab,ti OR 'heparin sodium 25, 000 units':ab,ti) AND 'dextrose 5%':ab,ti OR 'heparin sodium 25, 000 units in dextrose 5%':ab,ti OR 'heparin sodium 25, 000 units in sodium chloride 0.45%':ab,ti OR 'heparin sodium 25, 000 units in sodium chloride 0.9%':ab,ti OR 'heparin sodium 5, 000 units':ab,ti) AND 'sodium chloride 0.9%':ab,ti OR 'heparin sodium 5, 000 units in dextrose 5%':ab,ti OR 'heparin sodium 5, 000 units in sodium chloride 0.45%':ab,ti OR 'heparin sodium 5, 000 units in sodium chloride 0.9%':ab,ti OR 'heparin sodium b braun':ab,ti OR 'heparin sodium preservative free':ab,ti OR 'heparin subcutaneous':ab,ti OR 'heparin sulfate':ab,ti OR 'heparin sulfuric acid':ab,ti OR 'heparin sulphate':ab,ti OR 'heparina':ab,ti OR 'heparina leo':ab,ti OR 'heparinate sodium':ab,ti OR 'heparine':ab,ti OR 'heparine choay':ab,ti OR 'heparine novo':ab,ti OR 'heparinic acid':ab,ti OR 'heparitin monosulfate':ab,ti OR 'heparitin monosulphate':ab,ti OR 'hepcon':ab,ti OR 'hepflush-10':ab,ti OR 'hepsal':ab,ti OR 'inhepar':ab,ti OR 'inviclot':ab,ti OR 'lipo hepin':ab,ti OR 'lipo-hepin':ab,ti OR 'lipohepin':ab,ti OR 'liquaemin':ab,ti OR 'liquaemin lock flush':ab,ti OR 'liquaemin sodium':ab,ti OR 'liquaemin sodium preservative free':ab,ti OR 'liquemin':ab,ti OR 'liquemine':ab,ti OR 'menaven':ab,ti OR 'monoparin':ab,ti OR 'mucoitin polysulfate':ab,ti OR 'mucoitin polysulfate ester':ab,ti OR 'mucoitin polysulphate':ab,ti OR 'mucoitin polysulphate ester':ab,ti OR 'mucoitin sodium polysulfate':ab,ti OR 'mucoitin sodium polysulphate':ab,ti OR 'multiparin':ab,ti OR 'nevparin':ab,ti OR 'noparin':ab,ti OR 'panheparin':ab,ti OR 'panhepin':ab,ti OR 'panheprin':ab,ti OR 'parinix':ab,ti OR 'phlebotroy qps':ab,ti OR 'praecivenin':ab,ti OR 'pularin':ab,ti OR 'sodium heparin':ab,ti OR 'thrombareduct':ab,ti OR 'thrombo vetren':ab,ti OR 'thromboliquin':ab,ti OR 'thromboliquine':ab,ti OR 'thrombophlogat':ab,ti OR 'thrombophob':ab,ti OR 'thrombophob gel':ab,ti OR 'thromboreduct':ab,ti OR 'thrombosamine':ab,ti OR 'thrombosamine heparin':ab,ti OR 'thrombosamine heparine':ab,ti OR 'unfractionated heparin':ab,ti OR 'uniparin':ab,ti OR 'vetren':ab,ti OR 'vister':ab,ti OR 'vr 496':ab,ti OR 'vr496':ab,ti OR 'heparin':ab,ti | 127,616 results |
| 5 | 'percutaneous coronary intervention'/exp | 156,041 results |
| 6 | 'percutaneous coronary intervention':ab,ti | 79,127 results |
| 7 | #1 OR #2 | 8,285 results |
| 8 | #3 OR #4 | 257,538 results |
| 9 | #5 OR #6 | 156,041 results |
| 10 | 'randomized controlled trial':ab,ti OR 'controlled clinical trial':ab,ti OR randomized:ab,ti OR placebo:ab,ti OR 'clinical trials as topic':ab,ti OR randomly:ab,ti OR trial:ab,ti OR prospective:ab,ti OR retrospective:ab,ti | 5,064,312 results |
| 11 | #7 AND #8 AND #9 AND #10 | 1,151 results |

ab: Abstract; ti: Title; exp: Explode (Embase operator)
