# Supplementary material for: Comparison of bivalirudin versus heparin in percutaneous coronary intervention for patients with acute coronary syndrome: A meta-analysis
Source: Medicine (Baltimore). 2026 Sep 25;105(39):e50046. doi: 10.1097/MD.0000000000050046 (PMC13619192; doi:10.1097/MD.0000000000050046)
Supplement: Supplementary file 4 [file medi-105-e50046-s048.docx]

Supplementary Table 4 Search strategies and retrieved results in Cochrane Library database (search date: August 8, 2025)

| Cochrane Library | | |
| --- | --- | --- |
| No. | Query | Results |
| 1 | MeSH descriptor: [Hirudins] explode all trees | 442 results |
| 2 | (bivalirudin OR Hirudin OR BG 8967 OR BG-8967 OR BG8967 OR Hirulog OR Hirulog-1 OR Angiomax OR Angiomax RTU OR CTB-001 OR bivalirudin trifluoroacetate):ti,ab,kw | 895 results |
| 3 | MeSH descriptor: [Heparin] explode all trees | 6,248 results |
| 4 | (heparin OR Heparinic Acid OR Unfractionated Heparin OR Heparin, Unfractionated OR Sodium Heparin OR Heparin, Sodium OR Heparin Sodium OR alpha-Heparin OR alpha Heparin OR Liquaemin):ti,ab,kw | 13,507 results |
| 5 | MeSH descriptor: [Percutaneous Coronary Intervention] explode all trees | 8,889 results |
| 6 | (Percutaneous Coronary Intervention OR Coronary Intervention, Percutaneous OR Coronary Interventions, Percutaneous OR Intervention, Percutaneous Coronary OR Interventions, Percutaneous Coronary OR Percutaneous Coronary Interventions OR Percutaneous Coronary Revascularization OR Coronary Revascularization, Percutaneous OR Coronary Revascularizations, Percutaneous OR Percutaneous Coronary Revascularizations OR Revascularization, Percutaneous Coronary OR Revascularizations, Percutaneous Coronary):ti,ab,kw | 14,782 results |
| 7 | (randomized controlled trial OR controlled clinical trial OR Randomized OR placebo OR clinical trials as topic OR randomly OR Trial OR Prospective OR Retrospective):ti,ab,kw | 1,692,382 results |
| 8 | #1 OR #2 | 895 results |
| 9 | #3 OR #4 | 14,073 results |
| 10 | #5 OR #6 | 16,916 results |
| 11 | #8 AND #9 AND #10 AND#7 | 384 results |

ti: Title; ab: Abstract; kw: Keywords; MeSH: Medical Subject Headings; explode: Explode (Cochrane operator)
